# Feasibility of automated pre-screening for lifestyle and behavioral health risk factors in primary care

**DOI:** 10.1186/s12875-015-0368-9

**Published:** 2015-10-23

**Authors:** Gail L. Rose, Tonya A. Ferraro, Joan M. Skelly, Gary J. Badger, Charles D. MacLean, Tera L. Fazzino, John E. Helzer

**Affiliations:** 1 Department of Psychiatry, the University of Vermont, Burlington, VT USA; 2 Office of Research Administrative Services, Harvard University, Cambridge, MA USA; 3 Department of Medical Biostatistics, The University of Vermont, Burlington, VT USA; 4 Department of Medicine, The University of Vermont, Burlington, VT USA; 5 Department of Public Health and Preventive Medicine, University of Kansas, Kansas City, 10 USA

## Abstract

**Background:**

Screening of primary care patients for unhealthy behaviors and mental health issues is recommended by numerous governing bodies internationally, yet evidence suggests that provider-initiated screening is not routine practice. The objective of this study was to implement systematic pre-screening of primary care patients for common preventive health issues on a large scale.

**Methods:**

Patients registered for non-acute visits to one of 40 primary care providers from eight clinics in an Academic Medical Center health care network in the United States from May, 2012 to May, 2014 were contacted one- to three-days prior to their visit. Patients were invited to complete a questionnaire using an Interactive Voice Response (IVR) system. Six items assessed pain, smoking, alcohol use, physical activity, concern about weight, and mood.

**Results:**

The acceptance rate among eligible patients reached by phone was 65.6 %, of which 95.5 % completed the IVR-Screen (*N* = 8,490; mean age 57; 57 % female). Sample demographics were representative of the overall primary care population from which participants were drawn on gender, race, and insurance status, but participants were slightly older and more likely to be married. Eighty-seven percent of patients screened positive on at least one item, and 59 % endorsed multiple problems. The majority of respondents (64.2 %) reported being never or only somewhat physically active. Weight concern was reported by 43.9 % of respondents, 36.4 % met criteria for unhealthy alcohol use, 23.4 % reported current pain, 19.6 % reported low mood, and 9.4 % reported smoking.

**Conclusions:**

The percent endorsement for each behavioral health concern was generally consistent with studies of screening using other methods, and contrasts starkly with the reported low rates of screening and intervention for such concerns in typical PC practice. Results support the feasibility of IVR-based, large-scale pre-appointment behavioral health/ lifestyle risk factor screening of primary care patients. Pre-screening in this population facilitated participation in a controlled trial of brief treatment for unhealthy drinking, and also could be valuable clinically because it allows for case identification and management during routine care.

## Background

Among primary care (PC) populations, the prevalence of modifiable behavioral health problems is high [1]. For example, approximately one-quarter to one-third of PC patients internationally screen positive for unhealthy alcohol use on standardized instruments [2–4], about 20 % screen positive for smoking [5], and 5–10 % for depression [6–8]. In the U.S., 69 % of PC patients are overweight or obese [9], and about 30 % of PC patients report that they do not engage in any moderate to vigorous physical activity [10].

Screening of primary care patients for unhealthy behaviors and mental health issues is recommended by a variety of guidelines, including the United States Preventive Services Task Force (USPSTF) [11]. However, evidence suggests that provider-initiated screening is not routine practice [1, 12–17]. Screening is impeded by cost and time constraints, lack of administrative support, workflow incompatibility, limited provider awareness of validated screening instruments, provider discomfort or lack of confidence, lack of treatment resources for referring patients after screening, and stigma [18–22]. Furthermore, even when screening for behavioral problems is completed, there may be a gap in documentation in the medical record [23].

Ideally, behavioral health screening methods would be designed to address these barriers. Traditional paper questionnaires require data entry and are less practical as PC practice moves increasingly to electronic medical records (EMR). Computers or tablets can sync with the EMR and are more accessible to patients with limited literacy [24, 25]. Studies suggest that the majority of PC users have no difficulty using tablets for waiting room screening, although potentially vulnerable subgroups may need staff assistance [26]. Computerized administration and scoring allows for the use of skip patterns and customizability of the assessment. However, tablets are costly and require staff to distribute and collect them, and to field questions.

A less expensive and less staff-intensive method of EMR-compatible waiting room screening is to use dedicated telephones to access an Interactive Voice Response (IVR) system [27]. This method does not require reading, and assures privacy because responses are made with touch tones on a keypad. However, regardless of platform used, any screening that takes place in the waiting room requires staff effort and modification to clinic flow which is a threat to adoption and sustainability.

Alternatively, when screening is conducted prior to the patient’s arrival at the clinic, it can identify common preventive health issues ahead of time and thus remove responsibility for the screening from clinic personnel. Paper questionnaires delivered by surface mail have served this purpose for years; however, return rates can be low. EMR patient portals have great potential as a platform for pre-visit screening [28–31], but no published studies have evaluated their use for systematic behavioral health screening in PC.

Using IVR to screen for unhealthy behaviors and mental health issues before a visit may minimize the barriers of cost, privacy, literacy, EMR compatibility, and standardization of assessment. Our previous small-scale pilot study of an IVR screen demonstrated feasibility of the technology when offered on site at the time of a visit [27]. The current study aimed to improve upon this pilot study by conducting the screening prior to the office visit, with a larger target population at multiple practices, and with EMR integration. Patients identified during the screen were later referred to a brief alcohol intervention study. This paper reports the results of using this methodology for screening, and on the comparability of these screening results to those obtained by other studies using different methods. This evaluation of IVR pre-screening will inform future efforts to obtain reliable patient generated data that facilitate meaningful use of the EMR.

## Methods

### Recruitment procedures

Participant recruitment occurred from May, 2012 to May, 2014, in accordance with procedures approved by both the University of Vermont Committee on Human Research in the Medical Sciences and the University of Vermont Medical Center Department of Risk Management. Physicians (*N* = 40) from 8 PC clinics in an Academic Medical Center health care network recruited participants by mailing introductory letters to all patients aged 18 and older who were scheduled for routine, non-acute PC visits. The letters were generated two weeks prior to the scheduled appointments, and they provided an option to confidentially opt out of study participation. This step was required because the screening was conducted for research; in a non-research implementation, this step would not be necessary.

Any patient who had not opted out of the study received a call from a research assistant in the evening, three days before their scheduled clinic visit. If patients were not reached at the initial telephone call, they were called up to two more times on subsequent days, once in the afternoon, and once in the morning.

Patients were told that the study involved a 2-min automated telephone health screen that consisted of six questions (see Table 1), and that with their permission the answers could be sent to their EMR where they would be available for their PCP in time for their upcoming appointment. Patients who consented were then transferred directly to the IVR system to complete the six-item screen.

**Table 1:** IVR Screen script

Screen Item and Valid Responses	Brief Feedback for Positive Responses
*Pain*	
Are you having pain today?	Be sure to discuss your pain with your doctor at your visit. In addition, there are other pain management options at Fletcher Allen^†^ that your doctor may recommend.
No	
Yes*	
1–10 rating	
*Smoking*	
Do you smoke?	Please be sure to discuss smoking with your doctor at your visit. Quitting smoking is one of the best things you can do to improve your health. For additional help in quitting smoking, call the Vermont Quit Network at 1–800-QUIT-NOW
No	
Yes*	
*Drinking*	
IF MALE: About how many times in the past year have you had five or more drinks 1 day?	Please be sure to discuss drinking habits with your doctor at your visit. Your doctor may have recommendations about how alcohol can affect your health.
0	
1–365*	
IF FEMALE: About how many times in the past year have you had 4 or more drinks 1 day?	
0	
1–365*	
*Physical activity*	
How physically active are you?	Doctors recommend exercising for at least 30 min, 3 times a week. If you have concerns about your level of physical activity, please discuss them with your doctor at your visit.
Not at all active*	
Somewhat active*	
Very active	
*Weight*	
Are you concerned about your weight?	Be sure to discuss your concerns with your health care provider. He or she can evaluate your weight and make recommendations for how to maintain a healthy weight for you.
No	
Yes*	
*Mood*	
During the past 2 weeks, have you felt down, depressed, or hopeless?	Be sure to discuss your feelings with your doctor, who can help you decide if you need further treatment. There are mental health treatment options at Fletcher Allen that your doctor may recommend.
No	
Yes*	

*IVR* interactive voice response. Responses with * are considered positive

^†^Fletcher Allen refers to Fletcher Allen Health Care, the medical center these clinics were part of. In 2014, Fletcher Allen Health Care’s name was changed to University of Vermont Medical Center

### IVR screen development

The wording of the six screening items appears in Table 1. Items were chosen in consultation with participating providers and reflect influences of evidence-based guidelines, regulatory requirements, and the prevailing preferences of the providers. Only one item for each topic was chosen in order to keep the survey brief, yet reasonably broad.

#### Pain

The assessment of pain was included because of its status as the “5th vital sign” by the Veterans Health Administration [32]. Routine assessment and chart documentation of pain, including the use of a 10-point intensity rating, is a requirement in the Veteran’s Administration healthcare system and for health care organizations accredited by the Joint Commission (cf. Standard PC.01.02.07) [33].

#### Smoking

The USPSTF recommends that clinicians ask all adults about their use of tobacco products and provide tobacco cessation interventions for those who use tobacco [34]. This is a grade A recommendation.

#### Drinking

Unhealthy alcohol use was ascertained using the Single Alcohol Screening Question (SASQ) [35], as recommended by the National Institute on Alcohol Abuse and Alcoholism [36]. The USPSTF recommends screening adults for alcohol misuse, and providing brief counseling interventions to those engaged in risky or hazardous drinking (“B-level” recommendation) [37].

#### Physical activity

The USPSTF recommends that PC clinicians counsel patients about physical activity as a cardiovascular disease prevention measure, but to do so selectively rather than incorporate counseling into the care of all adults in the general population [38]. Others have advocated assessment of the frequency and duration of physical activity as a vital sign [10, 39]. However, self-reports of the intensity, duration, and frequency of exercise are typically overestimates [40]. For the purposes of this study, a qualitative self-report measure of activity level was chosen because medical circumstances may restrict a person’s ability to exercise at a specific intensity and frequency level, and thus clinical recommendations would be highly variable across respondents to the IVR-Screen.

#### Weight concern

Because objective assessment of weight would be obtained at the time of the visit, the IVR Screen assessed the patient’s own concern about their weight. This single item was written to be inclusive of patient concerns about being underweight or overweight. Providers collaborating on this study use such information for goal setting and treatment planning purposes. The USPSTF recommends screening all adults for obesity and offering or referring patients with a BMI of 30 kg/m2 or higher to intensive, multicomponent behavioral interventions [41]. Furthermore, the USPSTF recommends that PC clinicians counsel patients about healthful diet and physical activity as a cardiovascular disease prevention measure, but to do so selectively rather than incorporate counseling into the care of all adults in the general population [38]. Thus, the IVR-Screen item identifying weight concern may help a clinician select patients who might be most receptive to brief behavioral counseling.

#### Mood

The USPSTF recommends screening adults for depression, but only when appropriate staff-assisted depression care supports are in place [42]. The IVR-Screen item was a modification of the PHQ-2, a commonly used, validated screening tool for depression [43], and was considered appropriate because supports could be offered at the time of the patient’s PC visit 1 to 3-days after the screening.

Positive responses were followed by brief feedback and instructions to discuss the issue with the provider at their visit (see Table 1). Thus, the screen conveyed a message that the items represented topics that providers deemed important for discussion.

## Results

### Recruitment flow

**Fig. 1:**
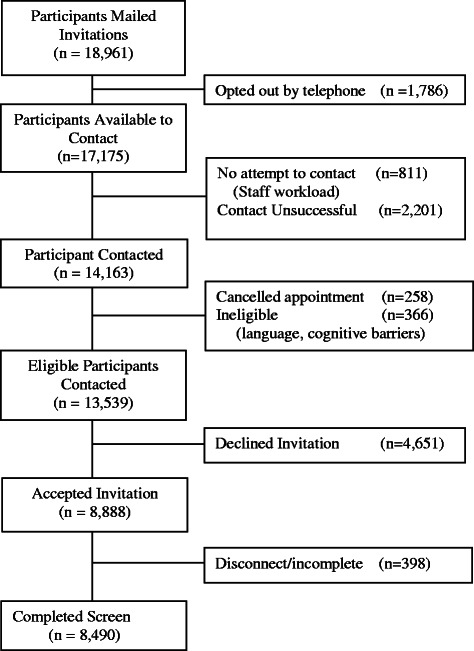
CONSORT diagram. This figure represents the flow of participant recruitment into the study

The flow of participants is presented in Fig. 1. Study invitation letters were mailed to 18,961 patients, 1786 (9.4 %) of which resulted in the patient opting out of the study. Of those who remained opted in, research assistants were able to contact 14,163 (83 %) patients in the 3 days prior to their scheduled visit. During the recruitment call, it was determined that some patients (*n* = 624) were ineligible for participation because their primary care provider (PCP) appointment had been changed, they were not proficient in English, or they had a cognitive or hearing impairment.

### Consent rates

The acceptance rate among eligible patients reached by telephone was 65.6 % (66 % for women and 65 % for men), and varied slightly across age groups. For 18–29, 30–44, 45–64 and 65+ age groups the consent rates were 63, 67, 68 and 62 %, respectively. Acceptance rates for the eight PC clinic recruitment sites ranged from 59 to 69 %. Almost all participants who accepted the screen completed it (8,490; 95.5 %).

### Patient demographics

Table 2 shows demographic characteristics of patients invited vs. enrolled in the study. With the exception of greater female representation, the sample demographics reflect the characteristics of adults residing in the Burlington, Vermont metropolitan area. In comparison with the PC patients invited to participate, participants were slightly older (75 % of the sample was over age 44 vs. 70 % of those invited), and more likely to be married (65 % vs. 61 %). Other demographics of the sample, i.e., gender, race, and insurance type, were representative of the patients who were sent invitations.

**Table 2:** Patient demographics

Characteristic	Invited to Participate (*N* = 18,961)	Completed Screen (*N* = 8,490)
% Female	57	57
Marital Status		
% single	22	19
% married/civil union	61	65
% divorced/separated	10	10
% widowed	7	6
% White	96	97
Age		
% 18–29	11	8
% 20–44	18	17
% 45–64	43	46
% 65 +	28	29
Insurance		
% Private or commercial	58	58
% Medicare	32	33
% Medicaid	9	8
% Self-pay	1	1
Education		
% ≤ High School Diploma or GED	NA	21
% Some College /Associates degree	NA	25
% Bachelor’s Degree	NA	30
% Master’s Degree/ PhD/MD/JD	NA	24

*NA* Not Available

### IVR-Screen results

The most commonly endorsed item on the IVR-Screen was inadequate physical activity (64.2 %), followed by weight concern (43.9 %), unhealthy alcohol use (36.4 %), pain (23.4 %), low mood (19.6 %), and smoking (9.4 %). Eighty-seven percent of the sample endorsed one or more item (87.6 % of women and 85.9 % of men), and 59.0 % endorsed multiple problems (60.1 % of women and 56.8 % of men). The most common combination was weight concern and inadequate physical activity: 34.4 % of respondents endorsed both of these items. This was the most common combination for both women and men (38.6 and 29.0 %, respectively).

**Fig. 2:**
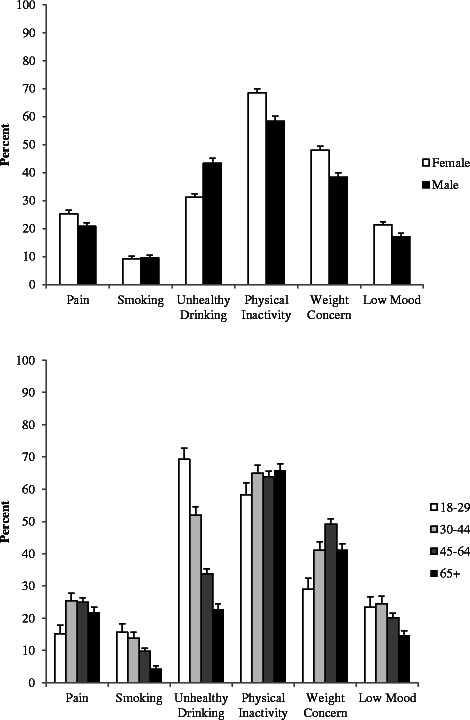
**a** Item endorsements by gender. This figure displays the percent of male and female participants who endorsed the six screening items, with 95 % confidence limits. **b** Item endorsements by age. This figure displays the percent of participants who endorsed the six screening items, with 95 % confidence limits, grouped by age category

Significant differences between men and women were observed for every item except smoking, as seen in Fig. 2a. Whereas more women than men endorsed the pain, activity, weight, and mood items, more men than women endorsed the alcohol item.

Endorsement percentages also differed by age. As shown in Fig. 2b, age was categorized as 18–29, 30–44, 45–64, and 65 years or older. In general, younger participants more frequently endorsed smoking and unhealthy drinking, but were more likely to be active. Older participants less often reported low mood, and middle aged participants reported more pain and concern about weight.

At the completion of the survey, participants had the option of sharing their screening results with their doctor, and 95 % chose to do so. Gender differences in opting to share were minimal; however, age comparisons showed that the sharing of results increased substantially with age, from 87 % for 18–29 year-olds compared to 98 % for those 65 and older.

## Discussion

This is the first study of fully automated, large-scale pre-appointment lifestyle and behavioral health screening of PC patients. Results indicate such screening is feasible. A majority of patients were willing to complete a pre-visit behavioral health screening by IVR as part of a voluntary research effort, even when given an option to opt out on the basis of not wanting to participate in research.

The findings demonstrated that most patients arrive to their doctors’ offices with lifestyle and behavioral health concerns. The rates of these concerns contrast starkly with the reported low rates of screening and intervention for such concerns in typical PC practice [1, 12, 14, 16]. In this sample, 87 % of respondents endorsed at least one screening item, which is comparable to previous studies that screened for multiple behaviors [1, 2]. Other studies did not report the specific behaviors that co-occurred, however. Here, weight concern and inadequate physical activity co-occurred in 34 % of participants.

Regardless of the number of other items they endorsed, the majority of patients reported being never or only somewhat physically active (64 %). It is difficult to compare this result to other studies in this area because physical activity has been measured in numerous ways that are only modestly correlated [44], and physiological measurement of participants’ activity levels has yielded much lower estimates than self-reported data [40]. However, population data from the Centers for Disease Control and Prevention (CDC) National Health Interview Survey indicated that approximately 40 % of US adults said they engaged in no leisure-time physical activity [45], and a PC chart review study found that about 30 % of patients self-reported no moderate to vigorous physical activity [10]. Importantly, the age and gender differences found in this study are consistent with nationwide surveys showing that more women than men reported limited physical activity, and that the percent of participants endorsing high activity levels decreased with age [40, 44].

Nearly half of the participants in this sample reported concern about weight (44 %). Consistent with existing literature, the frequency of weight concern was higher among women than men [46]. Prior research has shown that individuals’ subjective weight status (i.e., overweight, normal weight, underweight) does not always match their objective body mass index (BMI) classification and that there are consistent gender differences in the pattern of incongruences: Women’s misclassifications are more likely to be from normal weight women perceiving themselves as overweight, whereas men’s misclassifications are more likely to be from overweight men perceiving themselves as normal weight [47, 48]. The value of a subjective weight concern screening item is that it provides additional data to the PCP, beyond BMI alone, about the appropriateness of intervention on the issue.

Unhealthy alcohol use was endorsed by about a third of respondents overall, with men and younger patients meeting criteria at higher rates. These findings are consistent with existing literature on alcohol consumption in PC populations [2–4, 15, 49].

Reports of pain in this sample (23 %; mean rating 4.7) were somewhat lower than those reported in the literature. For example, a cross-national study from the World Health Organization reported that 34 % of PC patients experienced persistent pain at baseline assessment [50], and a study by Krebs et al. [51] found that 40 % of PC patients reported that pain was a reason for their visit. The mean rating for patients reporting pain in the Krebs et al. study was 6 on a 10-point scale [51].

About 20 % of participants endorsed low mood, which is higher than that shown in other studies. The reason for the relative over-identification of low mood in this sample is unclear, but may be due to the use of a single item with a dichotomous response option. In contrast, the Patient Health Questionnaire 2 Question Screen (PHQ-2) has a 0–3 frequency rating for each item, with scores of 3 or higher considered a positive result [43]. Consistent with other studies, more women than men in our study screened positive for depression.

Smoking was endorsed by 9.4 % of this sample, which is lower than other estimates of smoking in PC samples. Of note, Chittenden County, Vermont, has a smoking rate of 14 %, versus the national average of 21.2 % [52, 53]. Consistent with population statistics, this study data showed decreased smoking rates with age.

### Limitations

Approximately 9 % of patients opted out of the research upon receiving the study invitation letter. However, if IVR pre-screening were to be adopted as routine practice in the future, participation rates may be higher. That is, a screening questionnaire that is introduced by clinical personnel as standard protocol may be perceived by patients as having more legitimacy and relevance compared to an identical questionnaire that is presented by study staff as an optional research activity. This is an empirical question, however; it is possible that patients would be more motivated by the opportunity to participate in research. Nonetheless, some of the barriers to universal screening would remain, such as difficulties associated with systematic implementation, patient concerns regarding privacy and intrusiveness, and patient resistance to repeated assessments.

Approximately 17 % of participants could not be reached by phone prior to their appointment, which limits generalizability. Patient contact rates might be higher if patient health records specified cell as well as home numbers [54]. Also, if the call was a standard pre-visit clinical routine instead of a research effort, voicemail messages might be returned at a higher rate. Nonetheless, to capture a larger proportion of clinic patients in actual practice, clinics could offer additional optional screening platforms or processes such as patient report through an EMR portal. Patients who were not reached prior to the appointment could complete screening in the waiting room using an IVR or tablet.

The IVR-Screen did not use standardized screening instruments for every health domain because we were committed to single item screens. Brevity is of the essence in PC, and in wording the items we were responsive to input from the collaborating providers who preferred to have questions phrased according to their customary use. The single items used in this study can be considered pre-screen items, in that they identified patients who would benefit from more extensive evaluation by the physician in order to determine the best course of treatment. A second, more specific, screening step using validated instruments such as the AUDIT-C for alcohol misuse [55] would improve PCPs’ ability to interpret and prioritize screen-positive results.

### Strengths

In spite of the limitations noted above, the results of this study are valuable because they demonstrate the feasibility of IVR for pre-visit behavioral health screening and the willingness of patients to participate in such a process, even in a research context. The results showed rates of endorsement of screening items that are consistent with existing literature in spite of differences in screening methods. This convergence across methods supports the construct validity of the IVR-Screen [56]. Furthermore, the impact of this research is strengthened by the fact that the demographic characteristics of study participants closely match the characteristics of the patient population from which the sample was drawn, supporting the external generalizability of these results to the broader community.

## Conclusion

Managing behavioral health problems in the PC setting relies upon a system of case identification. Pre-screening is valuable because it allows identified individuals to participate in informed discussion with their physicians, who may determine that further assessment or treatment is needed. As this study demonstrated, IVR screening can be integrated with the existing technology and records management of a health care system, i.e., patient scheduling database, print and mail service, and EMR, and to the clinical flow of a PC practice. Such integration is rarely straightforward. The adoption of any new screening tool will require careful consideration of its content. The relative value of brevity, familiarity, and standardization of screening tools should be reflected in the items chosen. Finally, it is important to recognize that screening is simply the first step in the process of evaluating the need for treatment. Empirically supported, office-based brief interventions are available for some behavioral health conditions [11]. However; the finding that a majority of participants endorsed two or more concerns on the screen supports the need for interventions to address co-occurring conditions, for example, alcohol consumption and low mood.
